# IgY antibodies in human nutrition for disease prevention

**DOI:** 10.1186/s12937-015-0067-3

**Published:** 2015-10-20

**Authors:** Sandra Müller, Andreas Schubert, Julia Zajac, Terry Dyck, Christopher Oelkrug

**Affiliations:** 1 Fraunhofer Institute for Cell Therapy and Immunology (IZI), Perlickstraße 1, 04103 Leipzig, Germany; 2 IgY Immune Technologies and Life Sciences Inc., Thunder Bay, Canada; 3 Fraunhofer Project Centre for Biomedical Engineering and Advanced Manufacturing, McMaster University, Hamilton, Canada

**Keywords:** IgY, Egg yolk immunoglobulin, Nutrition, Oral passive immunization, Passive immunity

## Abstract

Oral administration of preformed specific antibodies is an attractive approach against infections of the digestive system in humans and animals in times of increasing antibiotic resistances. Previous studies showed a positive effect of egg yolk IgY antibodies on bacterial intoxications in animals and humans. Immunization of chickens with specific antigens offers the possibility to create various forms of antibodies. Research shows that orally applied IgY’s isolated from egg yolks can passively cure or prevent diseases of the digestive system. The use of these alternative therapeutic drugs provides further advantages: (1) The production of IgY’s is a non-invasive alternative to current methods; (2) The keeping of chickens is inexpensive; (3) The animals are easy to handle; (4) It avoids repetitive bleeding of laboratory animals; (5) It is also very cost effective regarding the high IgY concentration within the egg yolk. Novel targets of these antigen specific antibodies are Helicobacter pylori and also molecules involved in signaling pathways in gastric cancer. Furthermore, also dental caries causing bacteria like Streptococcus mutans or opportunistic Pseudomonas aeruginosa in cystic fibrosis patients are possible targets. Therefore, IgY’s included in food for human consumption may be able to prevent or cure human diseases.

## The avian immune system

To protect the host against invading microorganisms and exogenous antigens, the avian species have also developed an immune system similar to mammals. The avian immune system consists of primary and secondary lymphoid organs. Thymus, located in the neck and Bursa Fabricius, located adjacent to the cloaca, are primary lymphoid organs. Secondary organs are spleen, caecal tonsils, Harderian gland, bone marrow, lymph nodes, and various lymphoid tissues e.g. lymphoid tissues associated with mucosal surfaces (MALT); including bronchial-associated lymphoid tissues (BALT), gut associated lymphoid tissues (GALT) and conjunctival associated lymphoid tissues (CALT). The thymus is considered to be the primary lymphoid organ for T-cell differentiation and the antibody-synthesizing B-cells are formed in the Bursa of Fabricius, an organ which is unique in birds [1, 2]. The plasma cell proliferation and memory B-cells are situated within the spleen [3].

The avian immune system basically consists of two types: first the innate, non-specific system and second the acquired, specific system. The latter is mainly characterized by specificity and memory. This system can be distinguished into the cellular and non-cellular immune response, once again.

The cellular response can be defined as those cells that react with a high specificity to their specific antigen but not including the cells involved in antibody production. T-lymphocytes, as part of the cellular branch, recognize processed antigens on antigen-presenting macrophages. Among other tasks, T-cells are able to enhance or suppress the activity of B-cells, macrophages and T-helper cells. Further T-cells can directly destroy foreign organisms [4].

The non-cellular, also referred to as the humoral response, includes plasma proteins, e.g. immunoglobulins that passively circulate in blood or lymph, and antibody producing B-lymphocytes. These cells are formed in the embryonic liver, yolk sack and bone marrow. After maturation in the Bursa Fabricius, B-cells move to the blood, spleen, caecal tonsils, bone-marrow, Harderian gland, and thymus. T-helper cells are able to activate B-cells into plasmocytes. These plasmocytes are then able to secrete antibodies (immunoglobulins) which are highly specific for binding antigens. After re-exposing a chicken to the same antigen a memory effect occurs, involving an increased antibody production at a faster rate [5].

### Immunoglobulin classes in birds in comparison to mammals

Three classes of immunoglobulins in birds are known. First described is the mammalian IgG which is analogous to IgY and is mainly present in serum and egg yolk [6]. In previous studies, IgY has been called IgG because of its similar function and concentration in the serum. Nowadays, this terminology has been found to be incorrect, due to clear differences in molecular structure. Furthermore, recent studies describe the evolutionary relationship between avian IgY’s and human IgE [7]. In addition, IgY’s are also found in reptiles, amphibian and lungfish. The comparion of different Immunoglobulin classes are shown in Table 1.

**Table 1:** Basic comparison of the adaptive immune system between avian and mammals

Vertebrate class	Immunoglobulin isotype				
Mammals	IgM	IgD	IgG	IgE	IgA
Avian species	IgM		IgY		IgA

The adaptive immune system of avian and mammals is based on immunoglobulins. Birds produce three types of immunoglobulins (IgM, IgY and IgA), and the mammals five (IgM, IgD, IgG, IgE and IgA). In both cases the acquired immunity includes T-cell receptors (TCRs), polymorphic MHC class I and II molecules, primary and secondary lymphoid organs, rearrangement of the recombination- activating gene (RAG) and antibody class switch

Research has shown that other immunoglobulins including chicken IgA and IgM have similar molecular weight, structure and electrophoretic mobility compared to mammalian IgA and IgM.

IgY antibodies make about 75 % of all immunoglobulins. The serum concentrations of IgY, IgA, and IgM have been reported to be 5.0, 1.25, and 0.61 mg^.^ml^−1^, respectively [8].

In spite of functional homology between avian IgY and mammalian IgG, there are differences in molecular weight, structure and biochemical functions. They are composed of two identical heavy and light chains bound together with disulphide bonds. Furthermore, they own a variable antigen-binding-site and a constant highly conserved region. IgY’s are distinguished from IgG’s with major heavy chains and therefore a higher molecular weight. Moreover, avian IgY has a shorter and thus less flexible hinge region than IgG [9]. It has also been suggested that IgY’s have more hydrophobic molecules than IgG antibodies and also have a lower isoelectric point [10].

**Fig. 1:**
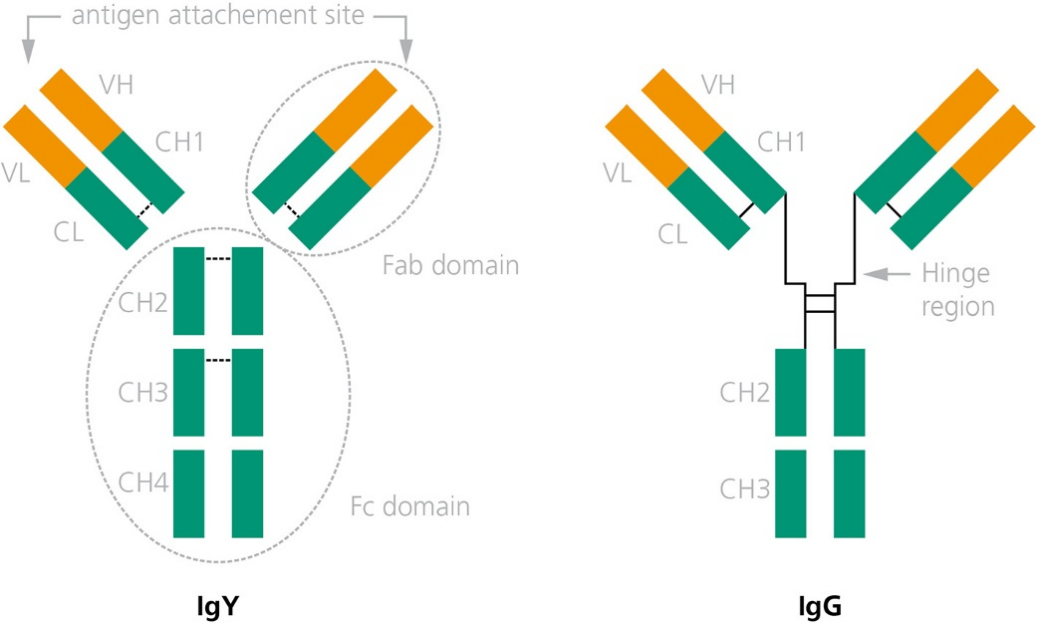
Structure of avian IgY versus mammalian IgG. Both molecules contain two heavy and two light chains, which consist of a variable domain (VH and VL) and four constant domains (CH1, CH2, CH3 and CH4), respectively. IgG has a longer hinge region, which makes it more flexible than avian IgY

IgY’s neither activate the complement system like IgG’s [11] nor interact with rheumatoid factors in Immunoassays [10]. The structural differences between IgY’s and IgG’s are shown in Fig. 1. Furthermore, IgY’s do not interfere with protein A or C. This may not simplify the purification but there are several methods for IgY extraction from the egg yolk.

The antibody diversity in chicken is distinguished from mammals and is based on gene conversion and somatic hyperconversion.

### Production of IgY’s starts with the immunization of chickens

IgY’s are an inexpensive and an alternative form of polyclonal antibodies. For immunization the hens were injected with specific antigens intramuscularly for several intervals. Antibodies are transferred from hen to the offspring via the latent stage of the egg. The immune-incompetent chick is able to resist various infectious diseases due to the innate immune system given from the hen. The transfer of IgY antibodies from maternal serum to the egg yolk is analogous to the cross-placental transmission in mammals [12]. During the last days of the embryonic development phase, IgY is transported across the yolk sac membrane into the embryonic bloodstream [13]. Recent studies show that the transfer of IgY from serum to egg yolk is a receptor-mediated process which allows a selective transfer of antibodies from the maternal serum [14, 15]. Research supported that a specific sequence (*His-Glu-Ala-Leu:* HEAL) in the FC-region and an intact hinge region are required for transport. Changes in this amino acid sequence inhibit IgY transport into the egg yolk. Roughly 4–6 days after inoculation, IgY’s can be detected in the egg yolk [16, 17].

The antigen dose significantly influences the immune response. Through renewed immunization the concentration of antibodies can be strongly increased in the egg yolk. This process avoids bleeding of animals, stress and permits the harvest of large amounts of antibodies. Furthermore, long-lasting high titre of IgY can be detected in egg yolk [18].

Schade et al. [19] published a review about all IgY extraction and purification methods. The most frequent used processes are with help of polyethyleneglycol [20], ammonium or sodium sulfate [21, 22]. After purification IgY’s show a high stability over a few months to a few years under specified conditions [19].

Gene-specific antibodies make the complicated multistep process for specific antigen synthesis redundant [23].

### Stability of avian IgY’s

For food fortification and the intestinal treatment to cure or prevent diseases, it is necessary, to improve the heat and pH resistance of IgY’s. Several studies have been conducted to evaluate the stability of these antibodies. The activity of IgY may be reduced by gastric conditions, particularly due to a low pH value [24]. Chicken antibodies are quite stable against digestive enzymes trypsin and chymotrypsin. Although there is a high loss of activity through Pepsin under low pH conditions in a short time [24, 25], IgY is stable at pH 4–9 and up to 65 °C in aqueous conditions. This resembles IgG, which is stable at pH 3–10 and up to 70 °C [26, 27]. However, the resistance of IgY to low pH conditions increases if high salt concentrations or stabilizing reagents e.g. sorbitol are present. Xylitol does not have such an effect on heat resistance [28]. Furthermore, the addition of sucrose increases the resistance against low pH ranges, heat and pressure [29]. The egg yolk may be able to stabilize IgY under low pH conditions and higher temperatures as well [30]. Several studies also describe protein modifications and coating-methods, respectively, and their positive effect against inactivation through digestion, heat or acidic conditions. Heat resistance of human IgG antibodies can be increased with help of Polyethyleneglycol-modifications [31]. Encapsulation on IgY’s in liposomes has been detected to stabilize antibodies against peptic hydrolysis under acidic conditions [32]. Furthermore, the protective effect of microencapsulation with chitosan–alginate on IgY during gastric passage *in vivo* has been evaluated [33]. IgY was reported to be stable for an extended durability for 14 weeks except at temperatures over 50 °C [30, 34]. Gujral et al. analyzed the IgY antibody stability during 78 weeks of storage at room temperature. In this case a combination with mannitol stabilized the IgY during the extended period [35].

### Advantages for using IgY’s

The production of IgY’s is a non-invasive alternative to current methods. The keeping of chickens is inexpensive and the animals are easy to handle. It avoids repetitive bleeding and pain of laboratory animals. Furthermore, it is also very effective. The IgY-titre in the egg yolk of immunized chickens remains very high for a long period of time [18]. One egg yolk contains more antibodies compared to the average isolated from the blood of immunized rabbits [36, 37].

IgY’s are able to prevent or cure human diseases as described previously. IgY’s are not able to pass the gastric barrier [28]. This offers various possibilities for passive immunization in the digestive system against pathogenic viruses and bacteria. But there are further application possibilities, e.g. in diagnostic tests or protein purification processes. IgY’s are less acid- and heat resistant than rabbit IgG [27, 38]. In 2002 Lee has shown that it is possible to stabilize the pH resistance with the help of sorbitol [28]. A further problem as well is the molecular resistance against proteolytic cleavage during the stomach passage. In 1993, Hatta and colleagues published, that IgY activity was reduced within a short time by pepsin, trypsin and chymotrypsin [26].

An advantage is that IgY’s neither activate the complement system nor bind to protein A and G, rheumatoid factors or cell surface [9, 36]. The phylogenetic distance between mammals and avian causes a high immune response of birds to mammalian antigens. This increases the binding specificity of IgY which is shown through immune diagnostics procedures like immunohistochemistry, ELISAs or immunofluorescence.

### IgY antibodies and bacteria

In 1984 Marshall and Warren firstly described *Helicobacter pylori*—previously named *Campylobacter pylori*—and its association with active chronic gastritis. They characterized the bacterium as a spiral, gram-negative organism with sheathed flagella. The organism is found in the human stomach and induces inflammation of the gastric mucosa [39]. It is known that *H. pylori* causes chronic gastritis, peptic ulcers, gastric mucosa associated lymphoid tissue (MALT) lymphoma and gastric cancer [40, 41]. Presently 70 % of all people in the world are infected with *H. pylori* [42]. But only a small number develop pathological symptoms. Previous studies showed that the eradication of *H. pylori* inhibits the reoccurrence of new gastric ulcers, cures several MALTomas and prevents peptic ulcers and gastric cancer [40, 43].

Nowadays the infection with the bacterium can be detected fast and often successfully medicated with specific antibiotic regimes which Wu et al. described in the Review of evidence-based therapies against *H. pylori*. But novel tools are necessary because there is a critical increase of antibiotic resistances.

Previous studies showed a positive effect of specific IgY’s against bacterial intoxications. The use of an oral therapy with IgY’s offers the possibility of passive immunization. There are several strategies of using IgY’s in host protection: a) inhibition of bacterial enzyme activity, b) toxin neutralization, c) blocking cell adhesion of microorganisms. The most effective way for passive immunization is using IgY produced as a response to previously selected characteristic bacterial antigens.

First trials for application of IgY’s in neonatal piglets were already conducted in 1998. Threatening enteric infections were treated with specific IgY antibodies against *E. coli* K88+ [44]. Later these therapeutic options were also validated with other *E. coli, Salmonella, Campylobacter* and Rotavirus strains [38, 45–48]. Enteric infections caused by *Salmonella* are another problem in chicken meat. Chalghoumi and colleagues published a summarized review about the need of alternative methods to prevent enteric infections in 2010. IgY’s may be a useful and attractive tool to passively immunize chickens and lower the infection rate in humans after consumption [5].

LeClaire et al. delivered chickens with staphylococcal enterotoxin B (SEB) from *Staphylococcus aureus*. After immunization they purified the egg yolk and treated rhesus monkeys with the extract. At the same time the monkeys were exposed to a lethal aerosolized dose of SEB. All animals which were treated with specific anti-SEB-IgY survived [49].

The actual transmission of *H. pylori* has never been clarified. The *H. pylori* urease-enzyme may be mainly responsible for colonization of human gastroduodenal mucosa. This enzyme allows the survival in the acidic gastric milieu. Urease creates a neutral microenvironment around the organism. It catalyzes the cleavage of urea to ammonia and carbon dioxide. This immediately increases the ph value and allows the implantation in the mucosa. The organism burrows into the mucosa to reach the epithelial cells where a less acidic environment is present [50]. Developed IgY’s against the whole-cell lysate of a *H. pylori* strain inhibit growth and urease activity of the organism *in vitro* [25]. In 2004 Horie and colleagues demonstrated the efficiency of specific anti-urease-IgY in designed drinking yogurt against *H. pylori* infections. They showed that the oral intake of highly specialized antibodies suppress the infection in humans. Due to urease inhibition the adhesion to the mucosa was also inhibited [51]. In 2009 Attallah and his group developed a reliable model for *H. pylori* infection in mice. Most cases of gastritis with inflammatory changes were detected. They developed IgY-antibodies against the cell lysate of a human pathogenic *H. pylori* strain in chicken and treated diseased mice with it. Results demonstrated that the passive immunized mice showed a significant lower degree of infection and gastritis than untreated animals [52]. Oral administration of preformed specific antibodies is an attractive approach against infections of the digestive system in humans and animals [9, 53].

### Monoclonal IgY-antibodies in gastric cancer therapy

*H. pylori* is a significant risk factor for the genesis of gastric cancer. As mentioned above, the eradication inhibits reoccurrence of gastric cancer. Changes in cellular signaling cascades lead to cancer development. Cells proliferate and grow uncontrolled quickly. The reasons for this are somatic mutations leading to permanently activated receptors and over-expression of receptors or ligands. Novel therapies addressed these targets. Monoclonal antibodies which are able to bind epitopes of the extracellular domain of growth factor receptors were able to prevent the receptor-ligand interaction and receptor dimerization. There are possibilities influencing the signal molecules, enzymes and intracellular receptors. The antibodies used at present are chimeric, humanized biological therapeutics [54]. It is conceivable that IgY’s administered orally cause no allergic reaction and can be used as a preventative therapy for gastric cancer caused by *H. pylori* or as a general prevention against inflammation caused by this bacteria. Additionally, because of its high stability it can be used as a food supplement, which increases the access of this therapeutic tool worldwide [55].

### Novel therapies against dental caries and periodontitis

Dental caries are a result of oral bacteria like *Streptococcus mutans* or *Streptococcus sobrinus.* To inhibit dental breakdown, a sugarless diet or use of fluoride is recommended. In addition to antibiotics or other antibodies, IgY’s can be applied against caries causing bacteria [56]. This was first demonstrated positively in rats [57] and then in humans as well [58].

In addition to caries, periodontitis also plays an important role in dental diseases. *Porphyromonas gingivalis* may be the main cause for periodontitis [59]. In 2007 it was demonstrated that IgY’s against a membrane-protein of *P. gingivalis* inhibits the development of a pathogen biofilm on the teeth surface and probably also following periodontal diseases [60].

### New target for egg yolk antibodies: *Pseudomonas aeruginosa*

Cystic fibrosis (CF), an autosomal recessive hereditary disease, affects mainly the lungs of patients among other symptoms. Causes are mutations in the gene encoding the cystic fibrosis transmembrane conductance regulator (CFTR) protein [61]. This leads to a reduced transport of chloride from exocrine cells and causes the development of viscous mucus in the bronchia. Chronicle lung infections by the opportunistic bacteria *Pseudomonas aeruginosa* (PA) are the main causes of morbidity and mortality in CF patients. In 2003, Kollberg and colleagues published that rinsing with a solution of specific anti-PA IgY in the evening may prevent the initial adhesion of the bacteria to the mucosal surface of the oropharynx in CF patients [62]. At the end of their study, all participants in the IgY treated group were still without chronicle PA infections. It was demonstrated that IgY could be used as a possible prophylaxis treatment in CF patients and thus reduces the necessity of frequent applications of antibiotics [62].

### IgY’s as a new treatment approach for Celiac disease

Celiac disease (CD) is an autoimmune disease triggered by the ingestion of gluten-containing grains in susceptible individuals. Here, wheat gluten and similar alcohol-soluble proteins are the causes responsible for the development of intestinal damage. The disease is characterized by a loss of absorptive villi and hyperplasia of the crypts. A gluten free diet is currently the only accepted treatment for celiac disease [63]. In 2012 Gujral and colleagues evaluated specific IgY antibodies against gliadin. As previously stated, purified IgY antibodies are extremely sensitive to gastric conditions and are rapidly inactivated by Pepsin under low pH ranges. The development of appropriate sugar protectants enables neutralization and the adsorption of gliadin in both *in vitro* and *in vivo* [35]. This could mean a revolution in the celiac disease treatment regime and harbors new hope for all affected persons.

## Conclusion

### Using IgY in disease prevention and outlook

Today IgY technology is a fast spreading field in life sciences. It may not be only a future vision anymore. IgY’s in nose spray, cosmetics, body lotions, functional food e.g. yogurt, powder supplement may be able to prevent or cure human diseases. The use of IgY antibodies offers the possibility for reducing antibiotics in the treatment of bacterial infections of the digestive system. In addition, egg yolk antibodies provide a new approach for attending *Candida albicans* and intestinal parasites as well. Furthermore, IgY treatment promises passive immunization in newborns and immune compromised patients. Patients during chemotherapy do not produce sufficient amounts of antibodies in response to vaccines. In this case passive immunization has provided new opportunities and an alternative to current treatment strategies. It may also be a new approach in therapy for chronic inflammatory bowel diseases e. g. crohn’s disease or ulcerative colitis. The local therapy with IgY antibodies in the digestive system could replace the current systemic treatment regime. Furthermore, IgY’s promise a new field for all basic prophylactic treatment strategies.

There is a high potential for IgY antibodies in the treatment of a variety of diseases and an even more prospective future.
